# Multivariate GWAS reveals shared genetic basis of common oral diseases

**DOI:** 10.3389/fgene.2026.1807175

**Published:** 2026-05-13

**Authors:** Xiyao Ma, Xiumei Zheng

**Affiliations:** 1 Department of Implantology, Stomatological Hospital of Xiamen Medical College, and Xiamen Key Laboratory of Oral Diseases, Xiamen, China; 2 Faculty of Science, National University of Singapore, Singapore, Singapore

**Keywords:** dental caries, GWAS, periodontitis, pulpitis, temporomandibular disorders, TMD

## Abstract

**Background:**

Oral diseases, including dental caries, periodontitis, pulpitis, and temporomandibular disorders (TMD), impose a substantial global burden affecting billions of individuals and costing an estimated USD 390 billion annually. Despite their frequent clinical co-occurrence, the extent to which these conditions share a common genetic basis remains unclear.

**Methods:**

We analyzed genome-wide association study (GWAS) summary statistics from up to 500,000 Finnish participants. Genome-wide and regional genetic correlations were quantified using High-Definition Likelihood. Genomic Structural Equation Modelling was applied to identify a latent common oral genetic factor (COF). A multivariate GWAS of the COF was conducted, followed by SuSiE fine-mapping. Integrative gene and pathway analyses were performed using cTWAS and MAGMA, and spatial mapping was conducted using embryonic tooth-germ atlases.

**Results:**

We identified extensive shared heritability across all four oral diseases. A latent COF captured the majority of this genetic overlap. Multivariate GWAS of the COF identified 104 genome-wide significant single-nucleotide polymorphisms aggregated into 96 independent loci, which were largely novel compared to single-trait analyses. Fine-mapping refined these to 53 high-confidence causal variants enriched in immune-regulatory and odontogenic pathways. CPSF1 and SLC20A2 emerged as top-ranked genes, with tissue-specific effects mapped to coronary artery and cultured fibroblasts, respectively. Spatial projection localized genetic risk to follicular and mesenchymal compartments, consistent with developmental tissue differentiation patterns.

**Conclusion:**

These findings reveal a shared and developmentally rooted genetic architecture underlying common oral diseases. The results highlight convergent molecular mechanisms and provide a foundation for precision-based, integrated prevention strategies that move beyond traditional single-disease frameworks.

## Introduction

Oral health is a major global public health concern. According to the Global Burden of Disease Study, oral diseases affect nearly 3.5 billion people worldwide, with untreated dental caries in permanent teeth being the most prevalent health condition globally (Bernabe et al., 2020). These conditions result in significant pain, impaired function, and economic burden, costing the global economy an estimated $390 billion annually (Peres et al., 2019). Among the most common oral diseases are dental caries, periodontal disease (PD), pulpitis, and temporomandibular joint disorders (TMD) (Asif et al., 2023). Each represents a distinct clinical entity with substantial public health impact. Dental caries is caused by bacterial acids that progressively demineralize tooth enamel and dentin (Selwitz et al., 2007). It remains the most prevalent health condition worldwide, affecting approximately 2.5 billion people with permanent dentition as of 2017 (Dye, 2017). PD is a chronic inflammatory disease that destroys the supporting structures of the teeth and is a leading cause of adult tooth loss (Hashim et al., 2025). Pulpitis can progress to pulp necrosis, periapical infection, or tooth loss (Pohl et al., 2024). TMD affects the jaw joint and associated musculature, resulting in chronic orofacial pain, joint clicking, and restricted movement (Schiffman et al., 2014).

Epidemiological and microbiological evidence supports the frequent co-occurrence of dental caries, PD, pulpitis, and TMD, suggesting shared pathogenic pathways (Silva et al., 2022). Large-scale cross-sectional analyses indicate that caries and periodontitis frequently affect the same dentition, with caries presence significantly increasing the odds of periodontal involvement (Baima et al., 2023). Pulpitis commonly develops as a downstream consequence of untreated caries or periodontal inflammation, reflecting a microbial and inflammatory continuum (Van Dyke et al., 2020). While causal relationships between PD and TMD remain uncertain, clinical data reveal notable comorbidity, potentially mediated by shared immunological and structural stress mechanisms (Wang et al., 2023). Despite previous genome-wide association studies (GWAS) identifying susceptibility loci for individual oral diseases and their frequent clinical co-occurrence, the extent of their shared genetic architecture remains poorly understood. This lack of integrative investigation is not due to a lack of importance but rather reflects the historical tendency of genomic studies to focus on single-disease outcomes. Given the overlapping microbial, inflammatory, and structural features of conditions such as dental caries, PD, pulpitis, and TMD, exploring their shared genetic underpinnings is both biologically plausible and clinically valuable (Rajasekaran et al., 2024).

This study aims to systematically interrogate large-scale GWAS data to identify genetic factors contributing to these prevalent oral conditions and to elucidate their shared genetic architecture. A deeper understanding of pleiotropic loci and converging biological pathways may offer mechanistic insights into oral disease pathogenesis. Ultimately, this work could inform holistic and precision-based prevention strategies, supporting efforts to reduce disease burden and promote both oral and systemic health.

## Methods

### Ethics statement

All data analyzed in this study were obtained from publicly available, de-identified sources. No new human participants or samples were recruited, and no identifiable individual-level data were accessed. Therefore, no additional institutional review board approval was required for the present study.

### Data source

We analyzed GWAS summary statistics from the FinnGen project (Kurki et al., 2023), a nationwide biobank integrating genomic data with longitudinal electronic health records from over 500,000 Finnish individuals. Four clinically and anatomically representative oral conditions were selected: dental caries (ICD-10: K02; 179,189 cases and 68,176 controls), reflecting degenerative lesions of the tooth’s hard tissue; PD (K05; 137,839 cases and 310,260 controls), involving chronic inflammation of the periodontal supporting structures; pulpitis (K04; 106,479 cases and 347,254 controls), indicating infection of the dental pulp; and TMD (K07.6; 20,799 cases and 479,549 controls), representing functional impairment of the masticatory system (FinnGen, 2025a). Together, these traits span the major anatomical compartments of the oral cavity and exhibit high prevalence in clinical settings, rendering them epidemiologically representative and biologically informative.

### Genetic correlation estimation via high-definition likelihood

To evaluate the genetic correlations ( $r_{g}$ ) among the four oral conditions, we applied High-Definition Likelihood (HDL) (Ning et al., 2020), a recently developed method for estimating $r_{g}$ using genome-wide association summary statistics. HDL offers an alternative to traditional approaches, such as linkage disequilibrium score regression (LDSC) (Bulik-Sullivan et al., 2015). While LDSC is widely used, it may yield imprecise estimates, especially when applied to traits with low SNP-based heritability. In contrast, HDL leverages a likelihood-based framework that accounts for linkage disequilibrium across the genome. This approach provides more accurate and stable estimates of genetic correlation, particularly for polygenic traits.

Using HDL, we estimated both global and local $r_{g}$ between each pair of traits. Local correlations were assessed across 2,468 approximately independent genomic regions, as defined by the HDL authors based on patterns of linkage disequilibrium in European populations. This dual-level approach enabled us to not only assess overall genome-wide genetic overlap but also identify specific genomic segments where shared genetic architecture may be concentrated.

### Multivariate genome-wide association analysis

Following the estimation of genetic correlations among the four oral phenotypes, we applied Genomic Structural Equation Modeling (Genomic SEM) (Grotzinger et al., 2019) to identify a latent dimension of shared genetic liability, which we termed the common oral genetic factor (COF). This framework leverages GWAS summary statistics to model the genetic covariance structure across traits and estimates Single Nucleotide Polymorphism (SNP) effects on the latent factor, yielding a multivariate GWAS. All downstream analyses were based on this multivariate GWAS of the COF.

To define genome-wide significant loci, we iteratively expanded ±500 kb windows around the most significant SNPs, merging overlapping regions until no genome-wide significant variants remained within a ±500 kb span (Zhou et al., 2022). To determine whether these loci were novel, we queried the GWAS Catalog using the Python package gwaslab (He et al., 2023). The search was conducted on 15th June 2025, and we checked for previously reported associations under the following EFO terms: dental caries (EFO_0003819), pulpitis (EFO_1001139), temporomandibular joint disorder (EFO_0005279), and periodontitis (EFO_0000649) (EBI, 2025).

### Causal variant finemapping

To pinpoint putative causal variants underlying the COF, we applied SuSiE (Sum of Single Effects) (Zou et al., 2022), a state-of-the-art Bayesian regression method for fine-mapping credible sets from GWAS summary statistics (Grotzinger et al., 2019). Finemapping was performed on loci identified from the multivariate COF GWAS using the 1000 Genomes Project European reference panel for linkage disequilibrium estimation (Koromina et al., 2025). Finemapping regions were defined using clumping with a ±500 kb window, an LD threshold of r^2^ < 0.001, and a genome-wide significance cutoff of P < 5 × 10^−8^. We retained only results from SuSiE models that achieved convergence, defined as a change in the variational lower bound (ELBO) of <0.001 between successive iterations (maximum 100 iterations). Variants with posterior inclusion probability (PIP) (Cui et al., 2024) > 0.8 were considered as likely causal (Li et al., 2025). This rigorous approach allowed us to distinguish candidate causal variants with high confidence across COF-associated loci.

### Gene and gene set enrichment analyses

To identify genes and biological pathways associated with the COF, we conducted gene-level and gene set enrichment analyses using MAGMA (Multi-marker Analysis of GenoMic Annotation) (Leeuw et al., 2015). This method aggregates SNP-level association signals into gene-level statistics and evaluates the enrichment of predefined gene sets—such as Gene Ontology biological processes—to uncover biological functions potentially relevant to COF.

### Causal TWAS to identify candidate effector genes

To further pinpoint genes with a likely causal role in the shared genetic architecture of oral diseases, we performed causal transcriptome-wide association studies (cTWAS (Zhao et al., 2024)) using the SuSiE-based framework. cTWAS integrates summary-level GWAS data with gene expression prediction models to estimate the effect of genetically regulated expression on the phenotype, while accounting for linkage disequilibrium (LD) and polygenicity (Bulik-Sullivan et al., 2015).

Gene expression prediction models were obtained from PredictDB (PredictDB, 2026), based on GTEx v8 across 49 tissues. GTEx v8 models are derived predominantly from European populations and are generally applicable to Finnish cohorts (which are of European ancestry), although minor differences in LD structure may introduce limited bias.

Compared with conventional approaches, SuSiE-based cTWAS improves specificity by modeling multiple causal variants per locus and computing PIPs for each gene, thereby reducing false positives arising from LD contamination. Genes with PIP > 0.8 were considered putatively causal for COF.

To enhance confidence in gene prioritization, we also examined the overlap between genes identified through MAGMA and those from cTWAS. Genes supported by both statistical enrichment and causal inference frameworks were considered higher-confidence candidates contributing to the shared genetic basis of oral diseases.

### gsMap analysis: genetically informed spatial mapping of cells for complex traits

Although the COF identified in this study was derived from a multivariate GWAS of four adult-onset oral diseases (dental caries, periodontitis, pulpitis, and temporomandibular disorders), previous evidence suggests that the genetic susceptibility underlying these conditions may be established during early embryonic tooth development (Zhao et al., 2024; Wentworth Winchester et al., 2022). To explore whether COF-associated signals exhibit spatial enrichment in embryonic dental tissues, we sought to trace their cellular origins during development.

We applied gsMap (Song et al., 2025) (genetically informed spatial mapping of cells for complex traits), a computational framework that integrates GWAS summary statistics with single-cell and spatial transcriptomics data to localize genetic signals to specific cell populations within spatial tissue architecture. This approach enables the projection of complex trait-associated genetic risk onto anatomically defined cellular contexts. In the present study, gsMap was used primarily to provide spatial and developmental context for the prioritized genetic signals, and the resulting maps were interpreted as supportive and hypothesis-generating rather than as direct evidence of adult disease mechanisms.

For the spatial reference, we utilized the publicly available spatiotemporal cell atlas of human embryonic tooth development published by Shi et al. (2024). This dataset comprises single-cell RNA-seq and spatial transcriptomic profiles from five human fetal tooth-germ samples collected at 17, 20, and 24 post-conception weeks. Both primary and permanent tooth germ tissues were included. In the original study, 11,218 quality-controlled single cells were profiled, and spatial transcriptomic data were generated from tooth sections of three donors spanning multiple developmental stages and dental compartments. We used this published atlas as the spatial reference for gsMap, while COF GWAS summary statistics were used as input to infer spatially enriched genetic signals.

**FIGURE 1:**
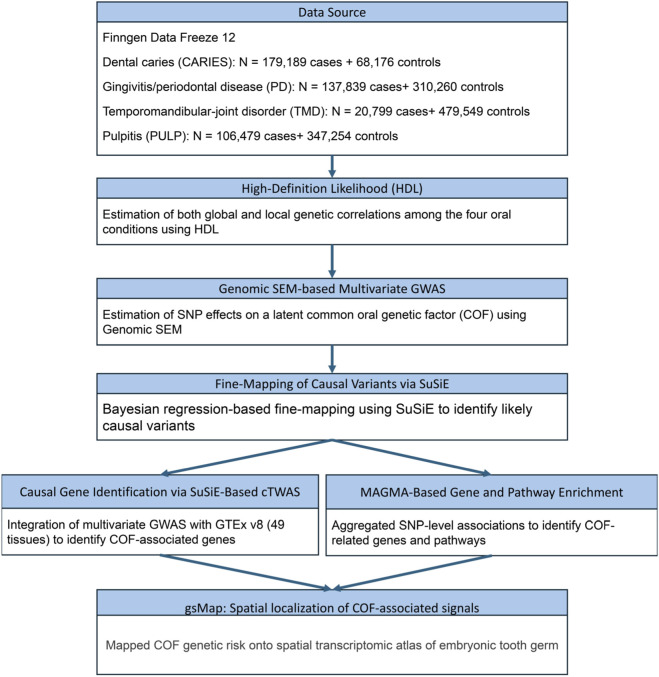
Flowchart of the analytical framework for identifying shared genetic mechanisms of common oral diseases.

The overall study design and analytical workflow are illustrated in Figure 1.

## Results

### Shared genetic architecture among oral diseases

**FIGURE 2:**
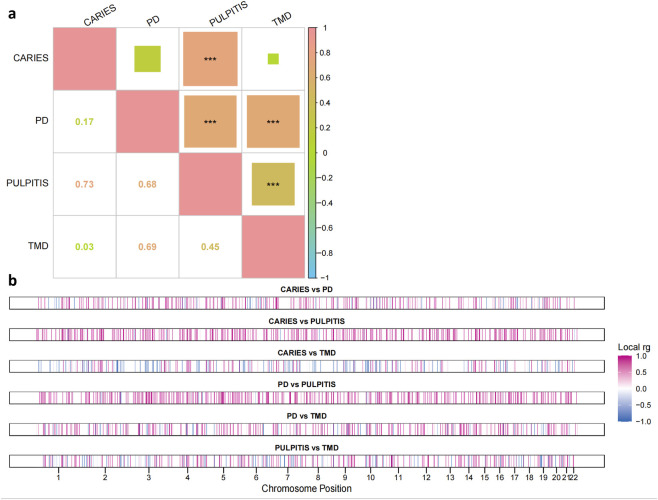
Genome-wide and local genetic correlations across four oral conditions. **(a)** Heatmap showing genome-wide genetic correlations ( $r_{g}$ ) among dental caries, periodontitis (PD), pulpitis, and temporomandibular disorders (TMD), estimated using high-definition likelihood (HDL). The color scale reflects the strength and direction of correlation, ranging from red (strong positive correlation) to green (weak positive correlation). Numerical values represent the $r_{g}$ estimates, and asterisks denote statistical significance: *p* < 0.05 (*), *p < 0.01* (**), and *p* < 0.001 (***). **(b)** Local genetic correlations (local $r_{g}$ ) between each trait pair across the genome. Each horizontal track corresponds to a pair of traits, with vertical bars indicating genomic regions across chromosomes 1 to 22. The color gradient represents the magnitude and direction of local $r_{g}$ , with blue indicating negative correlations and pink indicating positive correlations.

Significant $r_{g}$ among the four oral conditions were observed (Figure 2a). Caries and pulpitis exhibited the strongest genome-wide correlation ( $r_{g}$ = 0.73, *p < 0.001*), followed by PD and pulpitis ( $r_{g}$ = 0.68, *p < 0.001*), and PD and TMD ( $r_{g}$ = 0.69, *p < 0.001*). Moderate correlation was seen between pulpitis and TMD ( $r_{g}$ = 0.45, *p < 0.001*), while caries showed only relatively weak correlation with PD ( $r_{g}$ = 0.17) and TMD ( $r_{g}$ = 0.03).

To further dissect the genomic architecture underlying these correlations, we next examined local genetic sharing across the genome. Local genetic correlations revealed widespread regional sharing (Figure 2b). Among all trait pairs, caries vs. pulpitis and PD vs. pulpitis exhibited the highest density of strong positive local genetic correlations, with multiple enriched segments notably concentrated around chromosomes 5, 7, and 11. Caries vs. PD, PD vs. TMD, and pulpitis vs. TMD also showed widespread coverage of positive local $r_{g}$ signals, albeit with slightly reduced intensity and more neutral ( $r_{g}$ ≈ 0) regions. In contrast, caries vs. TMD was the only pair to exhibit multiple segments of negative local genetic correlation (local $r_{g}$ < 0).

### Multivariate GWAS identifies COF

**FIGURE 3:**
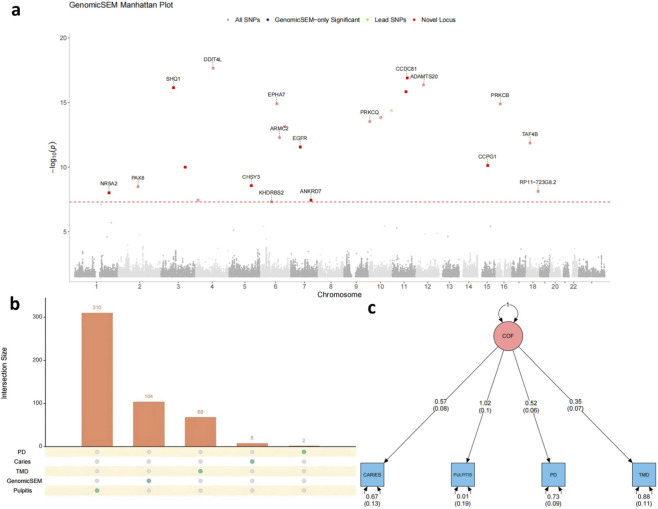
Multivariate GWAS identifies significant loci associated with the common oral genetic factor (COF). **(a)** Manhattan plot of the Genomic SEM-based multivariate GWAS. Each dot represents a SNP: blue dots indicate SNPs uniquely significant in Genomic SEM, green dots mark lead SNPs, and red dots highlight genome-wide significant loci. The red dashed line denotes the genome-wide significance threshold (P < 5 × 10^−8^). Annotated gene names correspond to lead SNPs at significant loci. **(b)** Bar plot showing the number of genome-wide significant SNPs identified in the multivariate Genomic SEM GWAS and single-trait GWASs for pulpitis (n = 310), temporomandibular disorder (TMD; n = 68), dental caries (n = 8), and periodontitis (PD; n = 2). Genomic SEM identified 104 significant SNPs, many of which were not detected by single-trait analyses, highlighting its increased power in detecting shared genetic architecture. **(c)** Structural equation model of the COF identified using Genomic SEM. The COF (pink circle) represents a latent factor capturing shared genetic liability across four oral conditions. The arrows indicate standardized factor loadings (standard errors in parentheses) from the COF to each trait: dental caries (0.57), pulpitis (1.02), PD (0.52), and TMD (0.35). Residual variances of each trait are also shown below the blue squares.

To investigate the shared genetic underpinnings of common oral conditions, we first applied structural equation modeling to genome-wide genetic correlations. This approach yielded a latent COF that loaded positively on all four traits—dental caries, pulpitis, PD, and TMD—indicating their convergent genetic architecture (Figure 3c). The standardized factor loadings were highest for pulpitis (1.02 ± 0.10), followed by dental caries (0.57 ± 0.08), PD (0.52 ± 0.06), and TMD (0.35 ± 0.07), suggesting that while each condition contributes uniquely, they share a substantial portion of genetic variance effectively captured by the COF.

Building on these findings, we next investigated the genome-wide distribution of SNP effects on the COF using GenomicSEM. Among the 7,994,986 SNPs analyzed, 104 reached genome-wide significance (P < 5 × 10^−8^) for the COF—compared to those identified in single-trait GWASs for pulpitis (n = 310), TMD (n = 68), dental caries (n = 8), and periodontitis (n = 2) (Figure 3b)—and were subsequently grouped into 96 independent loci. The Manhattan plot (Figure 3a) revealed multiple genome-wide associations, including lead SNPs located near genes with established roles in immune or developmental pathways, such as SHQ1, DDIT4L, EGFR, PRKCQ, ANKRD7, and TAF4B. From the 96 independent loci identified by the multivariate analysis, the majority represent novel associations at the trait level. Specifically, 93 loci were novel with respect to caries, 95 were novel for PD, and 94 were novel for TMD, as illustrated in Supplementary Figure S1. These findings highlight the trait-specific discovery gains enabled by Genomic SEM, capturing associations that were not previously detected by conventional single-trait GWASs.

We further evaluated the genetic insights gained from the multivariate model by performing SuSiE-based fine-mapping to prioritize putative causal SNPs. Based on convergence and a PIP > 0.8, a total of 53 high-confidence causal SNPs were identified (Supplementary Table S1), representing likely functional variants associated with COF.

### Gene-level and pathway-level functional annotation of COF

**FIGURE 4:**
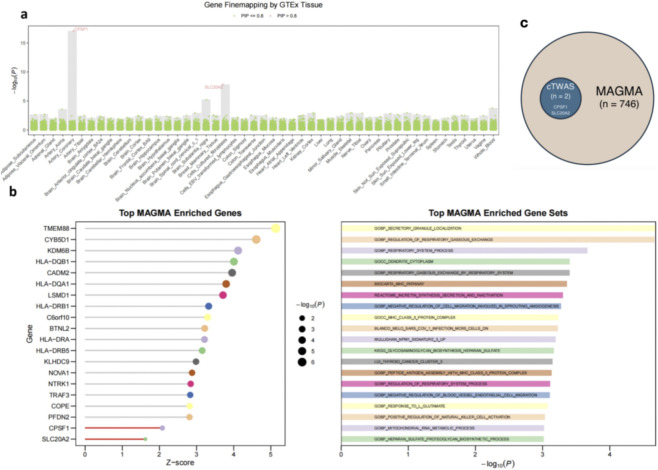
Gene and gene set prioritization for the common oral factor (COF). **(a)** Causal transcriptome-wide association study (cTWAS) results across 49 GTEx tissues. Red points indicate genes with a posterior inclusion probability (PIP) > 0.8 in specific tissues. Notably, CPSF1 in Artery–Coronary and SLC20A2 in Cells–Cultured fibroblasts were prioritized with both high PIP and significance. **(b)** Gene-level and gene set enrichment analyses using MAGMA. The left panel shows top genes with corresponding Z-scores and–log_10_(P) values, indicating gene-level enrichment. The right panel presents the most enriched biological pathways and processes, highlighting functions such as respiratory system regulation, immune signaling, and tissue development. Both CPSF1 and SLC20A2 reached nominal significance (p < 0.05), providing additional support for their involvement. **(c)** Overlap between genes prioritized by cTWAS and MAGMA. A subset-style Venn diagram showing the relationship between genes identified by cTWAS (PIP > 0.8) and those identified by MAGMA. cTWAS identified two genes (CPSF1 and SLC20A2), both of which are included among the 746 genes identified by MAGMA.

Aiming to uncover candidate genes and biological pathways involved in the COF, we began by performing a cTWAS across 49 GTEx tissues (Figure 4a). This analysis prioritized CPSF1 and SLC20A2 as tissue-specific causal candidates with high PIP (PIP = 0.999986 for CPSF1; PIP = 0.993742 for SLC20A2), with CPSF1 showing its strongest effect in coronary artery tissue and SLC20A2 in cultured fibroblasts. These results suggest that genetically regulated expression of these genes may causally influence COF in a tissue-specific manner.

To further validate these gene-level associations and explore their broader functional context, we applied MAGMA for genome-wide gene-level analysis (Figure 4b). Both genes reached nominal statistical significance (p < 0.05), lending additional support to their relevance. CPSF1 was found in pathways such as mitochondrial RNA metabolic process and negative regulation of cell migration involved in sprouting angiogenesis, while SLC20A2 appeared in heparan sulfate proteoglycan biosynthetic process and response to L-glutamate. Importantly, both CPSF1 and SLC20A2 were consistently prioritized across independent analytical frameworks, as they were identified by cTWAS (PIP > 0.8) and also included among genes highlighted by MAGMA (Figure 4c). Together, these approaches suggest that CPSF1 and SLC20A2 contribute to the shared genetic basis of oral diseases.

### Spatial mapping of COF-associated signals during embryonic tooth development

**FIGURE 5:**
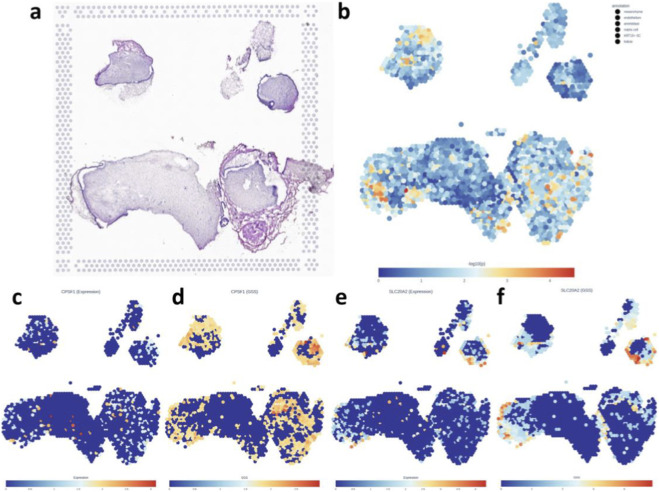
Genetically informed spatial mapping (gsMap) of the common oral factor (COF) using spatial transcriptomics data from human embryonic tooth germ. **(a)** Hematoxylin and eosin (H&E) (Shi et al., 2024)-stained sections show tooth germ development at various stages: *top left*, primary teeth at 17 weeks; *top right*, primary teeth at 20 weeks; *bottom right*, primary teeth at 24 weeks; and *bottom left*, permanent teeth at 24 weeks. At 24 weeks, both primary and permanent tooth structures are clearly identifiable. **(b)** Spatial distribution of COF enrichment scores (gsMap–log_10_P) across spatial transcriptomic spots in developing tooth germ tissue. Spots with warmer colors (red and orange) indicate higher genetic association signals. **(c–f)** Spatial transcriptomics visualizations of gene expression and genetically weighted spatial scores (GSS) for candidate genes CPSF1 and SLC20A2: **(c)** CPSF1 expression levels. **(d)** CPSF1 GSS highlighting significant enrichment scores. **(e)** SLC20A2 expression levels. **(f)** SLC20A2 GSS demonstrating marked enrichment.

To investigate the developmental origins of genetic risk, we utilized embryonic tooth germ tissues, which represent the earliest stages of tooth organogenesis. The histological reference of the sampled tissue is shown in Figure 5a.

Using gsMap, we projected COF GWAS signals onto spatial transcriptomic maps of embryonic tooth germ tissues (Figure 5b). A total of 1,849 spatial transcriptomic spots were profiled and annotated into six distinct cell populations within the developing human tooth germ, including mesenchyme, endothelium, ameloblasts, matrix cell, KRT15+ SC, and follicle.

Cell-type-level analysis revealed that follicle cells exhibited the highest proportion of significantly enriched spots (–log_10_P > 2; Supplementary Figure S2) and the strongest median enrichment across all annotated cell types (Supplementary Figure S3), which was further supported by Cauchy combination tests confirming statistically significant enrichment of COF signals across all compartments (P < 0.05), with the most pronounced signals in the follicle (P = 1.19 × 10^−3^) and mesenchyme (P = 3.88 × 10^−3^) regions (Supplementary Table S2).

To further investigate the two candidate genes identified in Figure 4, we examined their expression levels and genetically Gene Specificity Score (GSS) across the developing tooth germ (Figures 5c–f).

CPSF1 expression was primarily localized to the lower portion of the tissue map, with enriched expression concentrated in the lower-central and lower-right cell clusters (Figure 5c). In contrast, SLC20A2 expression appeared more diffusely distributed and less intense than CPSF1, with scattered expression hotspots predominantly located at the upper-right and lower-left edges of the tissue architecture (Figure 5e).

We next examined genetically GSS to determine the tissue and cell-type specificity of CPSF1 and SLC20A2 in the COF-associated developmental landscape (Figures 5d,f). CPSF1 exhibited its highest GSS values in the mesenchymal core of the permanent 24-week tooth germ, particularly within the dental pulp region that includes scattered endothelial and pericyte populations (Supplementary Figure S4). This pattern suggests a pulp-centric specificity for CPSF1, aligning with vascularized mesenchymal tissues that are central to early odontogenesis. A secondary signal was also observed in the inner mesenchyme of the primary 17-week germ. In contrast, SLC20A2 demonstrated its strongest GSS enrichment in epithelial-derived domains, including the outer enamel epithelium and stratum intermedium, as well as along the apical margin of the permanent tooth germ, where CD24^+^ and HOPX^+^ apical papilla-like stem cells are localized.

## Discussion

In this study, we uncovered a COF that underlies shared heritable risk across four major oral diseases—caries, PD, pulpitis, and TMD. Genetic correlation analyses revealed substantial genome-wide and regional overlap among these conditions, reflecting a partially convergent genetic architecture. Building on this shared liability, our multivariate GWAS identified 96 independent loci, most of which were novel relative to individual traits, demonstrating the enhanced discovery power of joint modelling approaches. Fine-mapping further refined these associations to 53 high-confidence causal variants. Integrating cTWAS results, we prioritized CPSF1 and SLC20A2 as top candidate genes, reinforcing their potential cross-trait relevance. Spatial mapping of COF signals onto embryonic tooth germ tissues traced the developmental origins of this risk to follicular and mesenchymal compartments, with gene-specific localization of CPSF1 to vascularized pulp mesenchyme and SLC20A2 to epithelial and stem-like domains. The inclusion of TMD within this shared factor is particularly notable, because TMD is anatomically and clinically distinct from caries, periodontitis, and pulpitis. Rather than implying identical pathophysiology across all four conditions, the COF more likely captures partial convergence at the level of host inflammatory regulation, craniofacial hard-tissue remodeling, and pain-related biological pathways, which may contribute to TMD susceptibility while remaining distinct from the classic tissue-destructive processes of tooth- and periodontium-centered disease.

Further insight into the underlying architecture emerged from local $r_{g}$ mapping, which pinpointed specific loci exhibiting coordinated risk between disease pairs. A prominent example is the 5q14.1–q14.3 region, which displayed particularly strong local $r_{g}$ between periodontitis and pulpitis (Supplementary Table S3). This locus contains immune-inflammatory and stromal genes such as AP3B1 (Shungin et al., 2019), BHMT, and HOMER1, and overlaps with enhancer-associated H3K27ac histone marks in periodontal fibroblasts, with conservation signals further supporting its regulatory signals (Cheng and Shen, 2025).

To sharpen causal inference, we applied the SuSiE fine-mapping model to the multivariate COF GWAS results. Among the 104 genome-wide significant SNPs identified, 53 variants were highlighted as likely causal with PIP > 0.8. These SNPs showed marked enrichment in immune-regulatory and odontogenic developmental pathways, suggesting that the pathogenesis of oral diseases is rooted in the molecular convergence of host defence and craniofacial morphogenesis (Figure 4b).

For instance, the locus near EGFR (rs77922120) exemplifies how COF-associated variants may enhance alveolar bone resorption by promoting osteoclastogenesis and inhibiting osteoblast differentiation (Schneider et al., 2009) (Supplementary Table S1). Similarly, a locus near PRKCQ (rs190156012) implicates adaptive immune signalling in COF risk, with evidence from gingival tissue showing that knockdown of its antisense lncRNA reduces NF-κB–driven transcription of key pro-inflammatory cytokines (Zhao et al., 2023). In the context of TMD, such shared loci may be especially relevant where inflammatory signaling, subchondral bone remodeling, and tissue stress responses intersect, which may help explain why a clinically heterogeneous disorder such as TMD still loads onto a common oral genetic factor.

cTWAS analyses identified CPSF1 and SLC20A2 as central gene-level contributors to the COF signal, underscoring their pivotal roles in mediating shared genetic susceptibility across oral diseases. Notably, these two genes may contribute to oral disease phenotypes through partially distinct biological routes. CPSF1, the largest scaffold subunit of the cleavage-and-polyadenylation specificity factor complex, is more plausibly linked to soft-tissue inflammatory pathology than to primary mineral defects, because altered 3′-end processing can reshape immune-relevant transcript usage and inflammatory signaling (Bergant et al., 2023; Evsyukova et al., 2013; Boreikaitė and Passmore, 2023). In the oral context, this mechanism may be most relevant to inflammation-dominant phenotypes, including pulpitis, periodontitis, and the inflammatory component of TMD. By contrast, SLC20A2 encodes the phosphate transporter PiT-2 and is more directly related to hard-tissue biology (Merametdjian et al., 2018). Experimental studies have shown that Slc20A2 deficiency disrupts dentin mineralization and impairs bone quality and strength, supporting a role in structurally mediated phenotypes such as deep caries progression, alveolar bone resorption, and osseous TMJ remodelling (Beck-Cormier et al., 2019). Taken together, CPSF1 and SLC20A2 may represent complementary mechanistic axes—predominantly inflammatory/regulatory for soft tissues and phosphate-handling/mineralization for hard tissues—through which shared genetic risk is manifested.

These interpretations are also consistent with emerging literature emphasizing the host–oral immune axis and systemic immunoregulatory contributions to oral disease, particularly in periodontitis, and support the view that shared oral genetic risk may act through both tissue-intrinsic and immune-mediated mechanisms (Ye et al., 2025; Ye et al., 2024).

These findings gain further biological plausibility from spatial transcriptomic analyses. Projecting COF signals onto embryonic tooth germ tissue maps revealed clear enrichment in the follicular and mesenchymal compartments, mirroring the known developmental fates of implicated cell types. Consistent with this distinction, CPSF1 is localized primarily to the vascularized pulp mesenchyme, a niche enriched in endothelial and pericyte clusters that depend on rapid mRNA remodelling for angiogenesis, providing a mechanistic link to inflammation-driven diseases like pulpitis and periodontitis (Liu and Moore, 2021). In contrast, SLC20A2 was concentrated in the outer enamel epithelium, stratum intermedium, and CD24+/HOPX+ apical papilla stem-cell zones—regions central to phosphate metabolism during enamel maturation and root elongation. Variants disrupting PiT-2 function in these zones could impair mineral integrity, predisposing individuals to deep caries, early root resorption, or condylar remodelling (Beck-Cormier et al., 2019; Serna and Bergwitz, 2020). However, these embryonic maps should be interpreted as providing developmental context rather than direct evidence of adult disease mechanisms. This conceptual distinction is particularly important for TMD, whose clinical manifestations often arise in postnatal joint, muscle, and pain-processing environments not fully captured by tooth-germ biology. Thus, the tooth-germ analyses are most directly informative for developmental susceptibility in caries, pulpitis, and periodontal tissues, while their relevance to TMD should be regarded as indirect and hypothesis-generating.

These mechanistic insights carry important implications for clinical practice. Unveiling the COF shifts the paradigm of precision dentistry beyond isolated disease models. Genetic screening for COF variants could guide the design of integrated preventive strategies that address multiple oral conditions simultaneously, echoing the “common-risk-factor” approach proposed for holistic patient care (Schwendicke and Krois, 2022). More broadly, our findings support a precision oral-health framework in which genetically anchored inflammatory susceptibility, tissue-remodelling propensity, and developmental vulnerability are considered jointly rather than disease by disease. This may be especially relevant for patients presenting with overlapping inflammatory and structural phenotypes, including those in whom periodontal, pulpal, and temporomandibular features. Methodologically, this study leveraged a biobank-scale Finnish cohort, the high-definition likelihood estimator, and a multivariate Genomic SEM framework layered with SuSiE, MAGMA, cTWAS, and gsMap, maximizing discovery potential.

Limitations of this study should be acknowledged. The Finnish founder population, characterized by a relatively homogeneous genetic background, may improve fine-mapping resolution; however, differences in allele frequencies and linkage disequilibrium (LD) structure compared to other populations may limit the generalizability of specific causal variant localization to non-Finnish ancestries (Kurki et al., 2023; FinnGen, 2025b). In addition, reliance on ICD-based phenotyping could introduce misclassification, and the lack of gene–environment interaction data may obscure important contextual influences on disease expression. Finally, the tooth-germ analyses provide developmental and spatial context but do not by themselves establish causal relevance in adult tissues. These considerations should be taken into account when interpreting causal inference, developmental localization, and the generalizability of the findings.

Future directions should focus on replicating these findings in diverse ancestry cohorts with finer clinical stratification, experimentally validating the roles of CPSF1 and SLC20A2, and integrating genetic data with oral microbiome and exposome profiles. Such efforts will be instrumental in constructing multi-omic risk models and deepening our understanding of gene-environment interplay in oral disease (Rajasekaran et al., 2024).

In sum, convergent genetic, functional, and spatial evidence supports a unified, developmentally rooted genetic basis for major oral conditions and highlights the promise of COF-informed strategies in advancing precision oral healthcare.

## Data Availability

The original contributions presented in the study are included in the article/Supplementary Material, further inquiries can be directed to the corresponding author.
