# Supplementary material for: Multivariate GWAS reveals shared genetic basis of common oral diseases
Source: Front Genet. 2026 May 13;17:1807175. doi: 10.3389/fgene.2026.1807175 (PMC13211857; doi:10.3389/fgene.2026.1807175)
Supplement: Supplementary file 1 [file DataSheet1.docx]

Figure List

**Supplementary Figure s1.**
**Counts of novel SNPs identified per trait.**
Bar plot showing the number of SNPs uniquely identified by the multivariate GWAS for caries (n = 93), periodontitis (n = 95), and TMD (n = 94), based on comparison with the GWAS Catalog.

**Supplementary Figure s2.**
**Enrichment of COF-associated signals across embryonic tooth cell types.**
Bar plot displaying the proportion of spatial transcriptomic spots with –log_10_(p) > 2 across annotated cell types, indicating follicle cells as the most enriched compartment.

**Supplementary Figure s3.**
**Distribution of COF signal enrichment across cell types.**
Boxplot of –log_10_(p) values for COF association across different cell types, showing the central tendency and variability of enrichment.

**Supplementary Figure s4.**
**Spatial distribution of annotated cell types during human embryonic tooth development.**
Spatial maps of primary (17, 20, 24 weeks) and permanent (24 weeks) teeth, showing anatomical localization of various cell types across developmental timepoints.


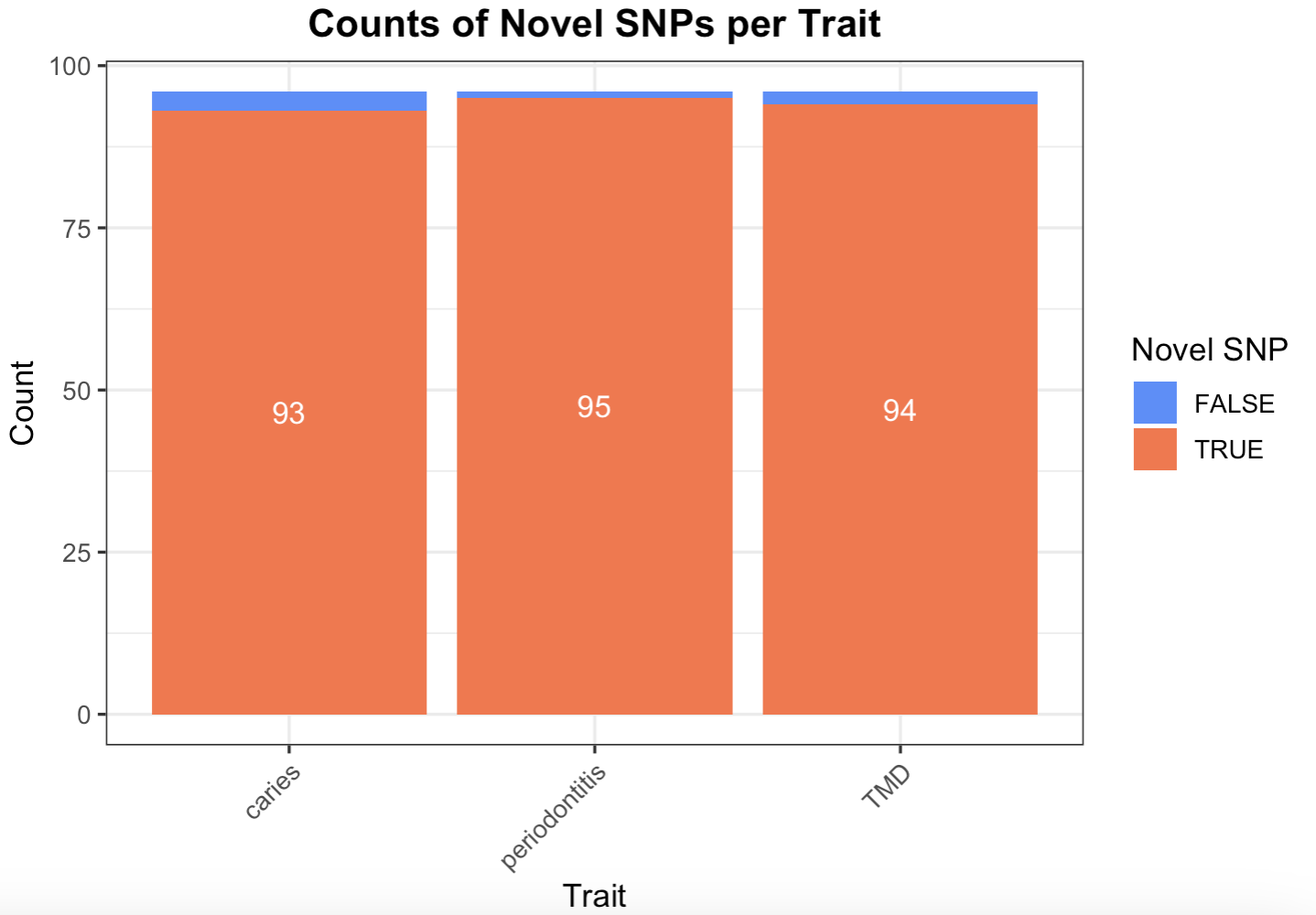


Figure s1.


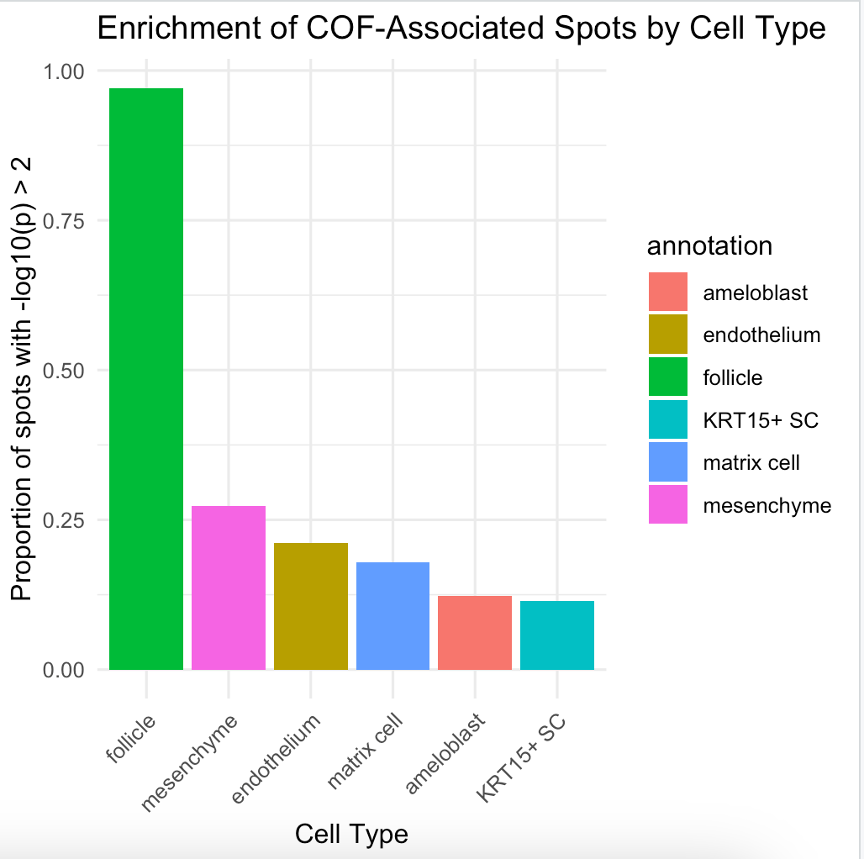


Figure s2.


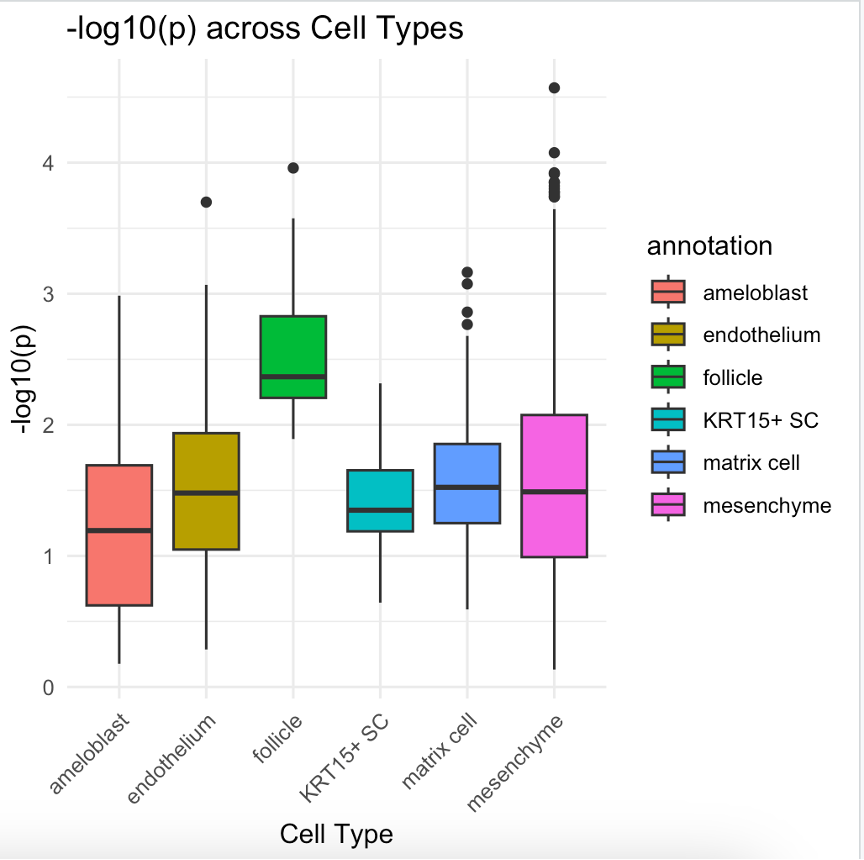


Figure s3.


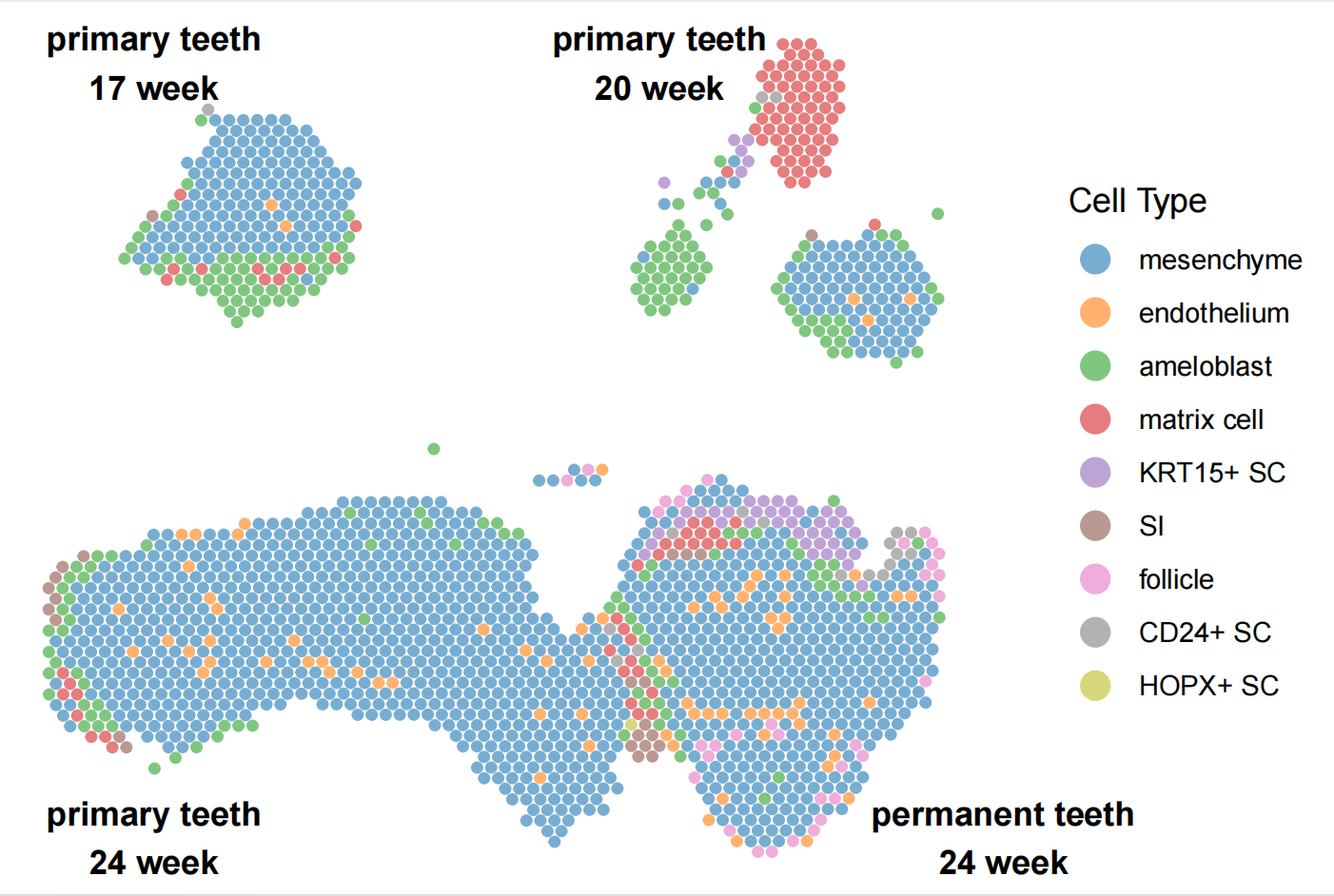


Figure s4.
